# Neurotropic Cutaneous Malignancies: Case Report on Keratinocyte Derived Malignancies of the Head and Neck With Perineural Invasion

**DOI:** 10.3389/fonc.2022.846278

**Published:** 2022-05-23

**Authors:** Grace Sora Ahn, Brian Hinds, Frederic Kolb, Amy K. Reisenauer, Seaver L. Soon, Ali R. Sepahdari, Kathryn B. Bollin, Soo J. Park

**Affiliations:** ^1^ Department of Dermatology, University of California, San Diego, La Jolla, CA, United States; ^2^ Department of Plastic Surgery, University of California, San Diego, La Jolla, CA, United States; ^3^ Private Practitioner, Kihei, HI, United States; ^4^ Private Practitioner, San Diego, CA, United States; ^5^ Diagnostic Neuroradiology, Scripps Clinic Medical Group, La Jolla, CA, United States; ^6^ Division of Hematology and Oncology, Scripps MD Anderson Cancer Center, La Jolla, CA, United States; ^7^ Moores Cancer Center, University of California, San Diego, La Jolla, CA, United States

**Keywords:** neurotropic cutaneous malignancies, non-melanoma skin cancer, cutaneous oncology, cutaneous squamous cell carcinoma, basal cell carcinoma, perineural invasion, immunotherapy, immune checkpoint inhibitors

## Abstract

**Background:**

The recent addition of immunotherapy as a treatment modality to surgery and radiation has vastly improved disease control for patients with keratinocyte-derived carcinomas (KCs) that are incurable with local therapies alone. With the advent of immune checkpoint inhibitors (ICPis) in non-melanoma skin cancers comes diagnostic and therapeutic challenges when considering treatment strategies for patients presenting with clinical perineural invasion (cPNI) of locally advanced KC of the head and neck.

**Objectives:**

We report four cases that convey the diagnostic and therapeutic complexity of managing patients with neuropathic symptoms from cutaneous neurotropic carcinomas of the head and neck. We also discuss an updated review regarding immunotherapies and perineural invasion within KC management.

**Conclusion:**

Patients presenting with symptoms suspicious for cPNI warrant an expanded diagnostic evaluation to correlate neurological findings with neurotropic spread of disease. While nerve biopsies can be precarious in sensitive areas, a history of skin cancer and clinical presentation suggestive of neurotropism may be enough to pursue timely management in the form of surgery, radiation, and/or systemic therapy given each patient’s individual priorities, comorbidities, and prognosis. When adding ICPi as a treatment modality for patients with disease not amenable to local therapies, the potential for immune-related adverse events must be considered. A multi-disciplinary review and approach to the management of patients with KC and cPNI is essential for obtaining optimal patient outcomes.

## Introduction

The revolutionary impact of immune checkpoint inhibitors on a wide range of malignancies has now expanded into the realm of advanced non-melanoma skin cancer. Achieving the impressive clinical benefits of immunotherapies for patients with high-risk and locally advanced cutaneous malignancies requires the engagement of surgical, radiation, and medical oncology specialists in highly complex decision making. Herein, we present four cases that highlight several diagnostic and therapeutic challenges when considering treatment strategies for locally advanced keratinocyte-derived malignancies of the head and neck with clinical perineural invasion.

Keratinocyte-derived carcinoma (KC) includes basal cell carcinoma (BCC) and cutaneous squamous cell carcinoma (CSCC)—ubiquitous cancers whose incidence is poorly documented due to exclusion from national and global tumor registries. While surgical excision of these cancers results in cure for most patients, in the setting of high-risk factors, such as >2 cm diameter, poorly differentiated histology, perineural invasion of nerves ≥0.1 mm, and tumor invasion beyond fat, there can be significant risk for local recurrence after surgery and/or radiation or for the development of locoregional metastases (1). Perineural invasion (PNI) is a rare complication of KC that involves tropism of tumor cells extending along the tissue stroma of the nerve sheath. Incidental PNI (iPNI) is found upon histological examination in the absence of symptoms. Clinical PNI (cPNI) is diagnosed radiographically and/or by the presence of sensory disturbances or motor deficits correlating with malignant neurotropism involving large caliber nerves. Radiographic definition of PNI involving the head and neck is described anatomically by a zonal classification system applied to magnetic resonance imaging (MRI) (2, 3). The mechanism of PNI involves migration along the nerve trunk within the perineural space that, in turn, activates numerous signaling pathways involving trophic factors, extracellular matrix adhesion proteins, and regulators of chemotaxis (4–9). In both BCC and SCC, for example, the presence of neural cell adhesion molecules may help determine such tumor aggression and increased levels of nerve growth factor, and TrkA, B, and C may reflect unique survival pathways (10). Emerging models of PNI suggest that interactions between tumor cells and nerves not only induce tumor migration but also stimulate axonogenesis and neurogenesis, which leads to both the enlargement and increased nerve density, respectively, around neurotropic malignancies (10–12).

Keratinocyte carcinomas with PNI are uncommon relative to the overall incidence of KC, with estimates of incidence rates of PNI in CSCC ranging from 2.5% to 14% and in BCC ranging from 0.18% to 10% (13–16). BCC with PNI independent of other high-risk features, including large diameter, aggressive histologic subtype, deep tumor invasion, and location on the face, does not appear to correlate with worse prognosis (17). However, CSCC with either iPNI or cPNI is associated with an increased risk for nodal and distant metastases, while patients with cPNI have worse recurrence-free and disease-specific survival with a 30% risk of death (18). Features associated with higher incidence of cPNI include male sex and previous history of skin cancer, whereas immunosuppression, lymph node involvement, and extranodal extension are independently associated with worse outcomes for patients with high stage primary CSCC (19, 20).

Diagnosis of PNI can be missed despite reliable techniques including histopathological examination, clinical examination evaluating neuropathic symptoms, and diagnostic imaging, such as high-resolution MRI (21–24). Recognizing the presence of PNI early may influence treatment decisions, which can in turn improve patient outcomes that have historically proven to be poor due to increased rates of disease recurrence and increased morbidity and mortality, especially in the setting of CSCC of the head and neck (HNCSCC) (18, 25–27). Location of cPNI can be inherently morbid, such as with perineural outgrowth into cerebral nerves resulting in severe pain or neurological disturbances such as cranial neuropathies (21, 22, 28). Invasive biopsies of neurologically sensitive areas present a diagnostic challenge; thus, a greater understanding of the association between neurotropism, symptomatic presentation, extent, and morbidity of disease is needed for physicians to best manage patients with the varying degrees of perineural spread. Treatment of patients with KC and PNI can include surgical excision, definitive and/or adjuvant radiation, concurrent chemoradiotherapy, hedgehog growth signaling pathway inhibitors for unresectable BCC, and, in the case of unresectable or advanced CSCC and BCC, immune checkpoint inhibition (ICPi) targeting the [programmed cell death protein 1 (PD-1) and its ligand L1 (PD-L1). For patients with cPNI and locally advanced KC of the head and neck, many of whom have had prior treatment with surgery and/or radiation, the potential for surgical disfigurement and/or exacerbation of underlying medical comorbidities with systemic therapies is a common confounding factor and should prompt multi-disciplinary review for individualized treatment planning.

In the four cases that follow, we seek to convey the complexity of therapeutic decision making for patients presenting with neuropathic symptoms from cPNI in KC of the head and neck and to highlight current gaps in knowledge that may warrant further research.

## Case Presentations

### Case 1

**Figure 1:**
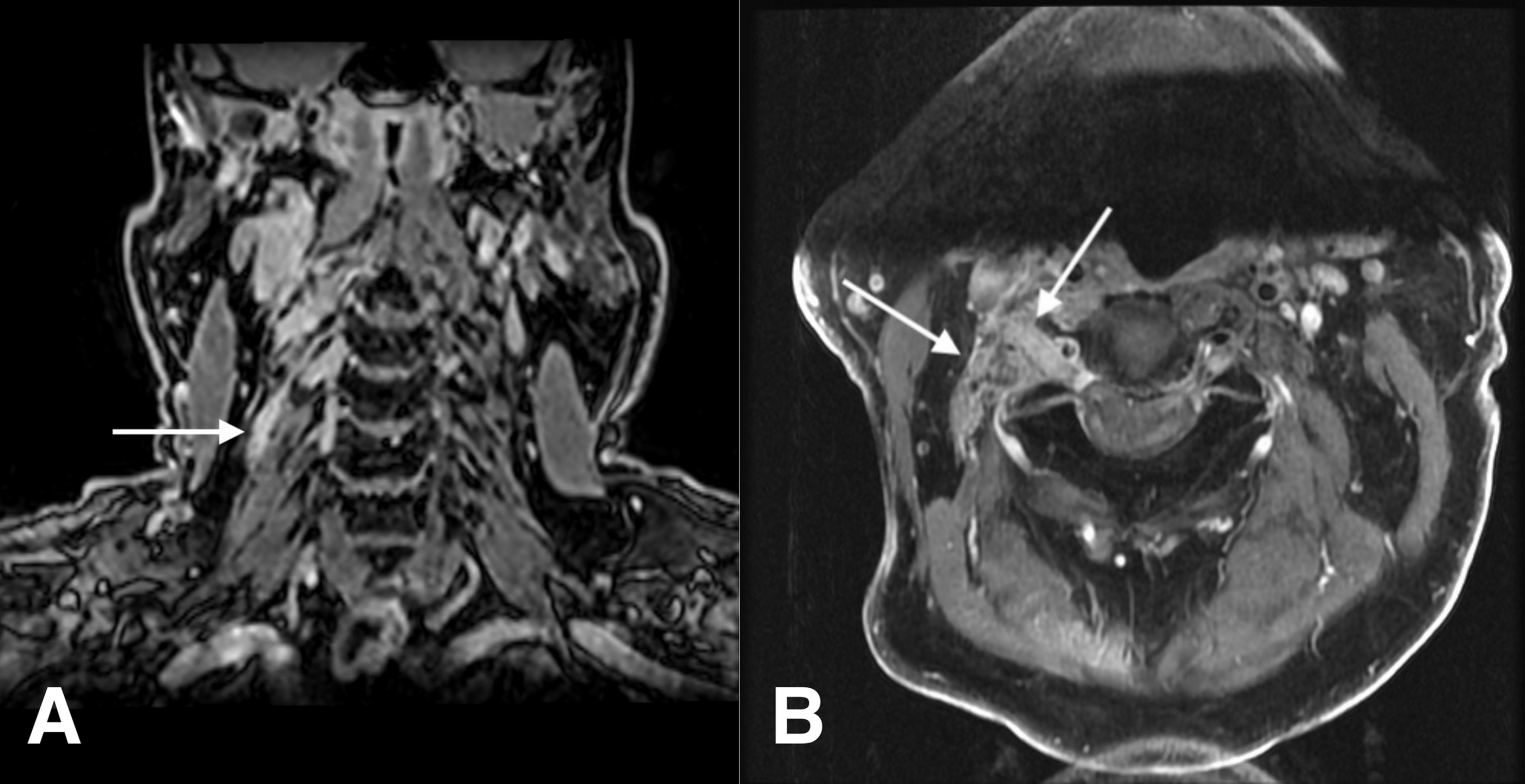
Case 1: a 74-year-old man with squamous cell carcinoma. **(A)** Coronal fat-suppressed contrast-enhanced T1-weighted image shows a thickening, abnormally enhancing right C4 nerve (arrow). **(B)** Axial fat-suppressed contrast-enhanced T1-weighted image shows thickening and abnormal enhancement of the right C3 nerve, from the dorsal root ganglion through its course through the posterior cervical space (arrows).

A 74-year-old man with a history of multiple cutaneous SCC of the head and neck presented to the clinic in June 2021 with severe neuropathic pain of the right neck and scalp. In October 2020, the patient noted gradually increasing pain in the right shoulder that did not improve with physical therapy. After 2 months of increasing numbness that extended to the right anterior upper chest wall and eventually to the jaw, MRI of the neck and shoulder in February 2021 showed asymmetric enhancement of the right C4 nerve just distal to the C3–C4 neural foramen (**Figure 1**). At this time, he was noted to have SCC of the right post-auricular region and vertex scalp and underwent Mohs microsurgery in February 2021. There was no histological evidence of PNI in the examined frozen sections. Neuropathy subsequently progressed further until the patient noted a right supraclavicular lump in April 2021. The right anterior deep neck mass was excised by head and neck surgery and diagnosed as a lymph node, associated with invasive SCC and without extranodal extension. MRI imaging in May 2021 revealed thickening of the right C3 and C4 nerves, with asymmetric contrast enhancement. After hematology oncology consultation, the patient was referred to radiation oncology to consider adjuvant radiation and to neurosurgery for cervical spine nerve root biopsy for suspicion of PNI of SCC. Options for treatment of high-risk SCC in the absence of gross or radiographic disease initially included adjuvant radiation to the post-auricular and supraclavicular regions versus ICPi, but radiation was ultimately not recommended due to difficulty in delineating the radiation field after rapidly progressive neuropathy. In June 2021, the patient underwent biopsy of the C3 nerve root with pathology demonstrating perineural SCC. Pembrolizumab 400 mg intravenously (IV) every 6 weeks was initiated. After the first dose of immunotherapy, the patient’s neuropathic pain worsened, and he developed clinical involvement of several right-sided cranial nerves, including the vagal and hypoglossal nerves, with vocal cord paralysis and aspiration. In August 2021, a gastrostomy tube was placed, and high-dose palliative radiation was delivered to the at-risk cervical spinal canal, involved neck, and all involved and at-risk cranial nerves up to the skull base. Pain of the right neck region improved until recurrence in October 2021, with CT neck revealing new right paravertebral and upper chest wall soft tissue masses. Carboplatin (AUC 5) IV and paclitaxel 80 mg/^m2^ IV were added to pembrolizumab. The patient tolerated three cycles of chemoimmunotherapy without clinical disease progression; however, he contracted severe acute respiratory syndrome coronavirus 2 (SARS*-*CoV*-*2) in January 2022 and died 1 month later from respiratory failure attributed to coronavirus disease 2019 (COVID-19) pneumonia.

### Case 2

**Figure 2:**
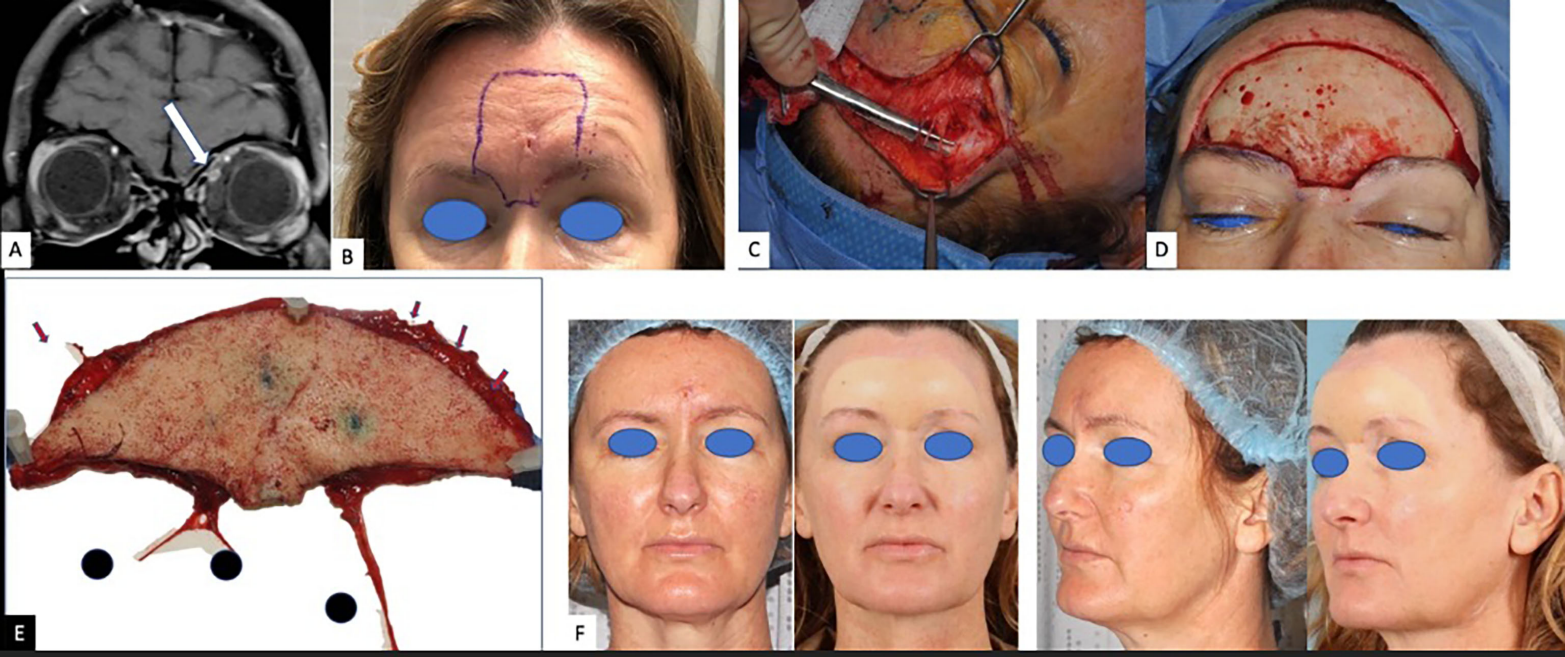
Case 2: a 50-year-old woman with basosquamous carcinoma. **(A)** Coronal fat-suppressed contrast-enhanced T1-weighted image shows asymmetric enhancement along the superior orbit (arrow), adjacent to the superior rectus muscle. **(B)** Pre-operative forehead recurrence. **(C)** Intra-operative dissection of the distal sensitive branches of the forehead sensory nerves. **(D)** Defect of the forehead after resection of the recurrence with 2 cm margins. **(E)** Pathological sample. Red arrows showing the tagged distal sensory branches. 1 = right supraorbital nerve; 2 = right supratrochlear nerve; 3 = left supraorbital nerve dissected until its entrance in the superior orbital fissure. The left supratrochlear nerve is missing and was dissected separately as interrupted by a pre-operative biopsy. **(F)** Pre - and 14-month post-operative clinical photos. Post-operative proton radiotherapy spared the reconstructed forehead and focused on the retro-orbital and skull course of the supratrochlear nerve including the Gasser nerve.

A 50-year-old woman presented to the clinic with headaches subsequent to the complete resection of a central left forehead BCC in April 2019. Histopathology was notable for admixed features including nodularity and a focal infiltrating proliferative pattern with one focus of PNI identified within a 0.05-mm caliber nerve fiber and negative margins. As the patient’s headaches increased in severity, pain management strategies including lifestyle adjustment, acupuncture, and migraine therapy proved ineffective. She eventually developed intermittent severe pain above the left eyebrow followed by numbness extending inferiorly to the left upper eyelid. She was treated with antibiotics for a presumed infection when swelling and drainage were noted. Symptoms did not abate and ultimately a punch biopsy in August 2020 demonstrated invasive, well-differentiated, and partially cystic SCC, 1.2-mm deep without overt perineural, perineurium, endoneural, or angiolymphatic involvement identified. MRI of the face showed nodular enhancement along the course of the left V1 frontal nerve branch, while MRI of the brain noted concern for extensive perineural spread along the superior medial aspect of the left orbit. The perineural spread (PNS) along V1 seemed not to extend beyond zone 1. Nerve biopsy of the left supraorbital nerve identified multifocal PNI, and tertiary comprehensive pathology review rendered a diagnosis of basosquamous carcinoma. Multidisciplinary review was held at three separate cancer centers, with two favoring immunotherapy due to high response rates in CSCC and anticipated disfigurement from surgery, while the third favored surgery followed by radiation as a curative intent treatment plan that provided microscopic definition of the extent of disease. Ultimately, the patient decided upon surgery and radiation for its curative potential and against anti-PD-1 immunotherapy due to potential toxicity. Surgery involved wide margin resection of glabellar SCC with exhaustive selective neural microdissection to assess precisely the clinical and incidental neural invasion beyond the surgical resection. All small and large nerve branches were identified and tagged at the margins. Small branches were traced for at least 5 mm. Large V1 nerve trunks (bilateral supratrochlear and supraorbital nerves) were dissected for at least 3 cm in their intra-orbital cavity course. The right V1 and left supratrochlear branches had a normal macroscopic appearance, and their dissection was not continued beyond the 3 cm. The left supra-orbital nerve was macroscopically enlarged in accordance with pre-operative MRI findings (**Figure 2**). Its selective microdissection was continued until its entrance in the superior orbital fissure. At this level, the nerve had a normal diameter. A sentinel node biopsy was included, and a total of four sentinel lymph nodes were resected. Histopathology revealed invasive poorly differentiated SCC, perineural spread (PNS) involving the supraorbital nerve with clear close margin, no PNI along the small nerve branches, and negative lymph nodes. Upon final histopathological assessment, she was diagnosed with basosquamous carcinoma of the forehead with macroscopic V1 neurotropism bilaterally. Following surgery, the patient was treated with intensity-modulated proton therapy (IMPT) to the left V1, including zone 1 and 2 and glabella to better spare optic structures. At 1-year follow-up, her typical trigeminal headaches were absent, and she remains disease free.

### Case 3

**Figure 3:**
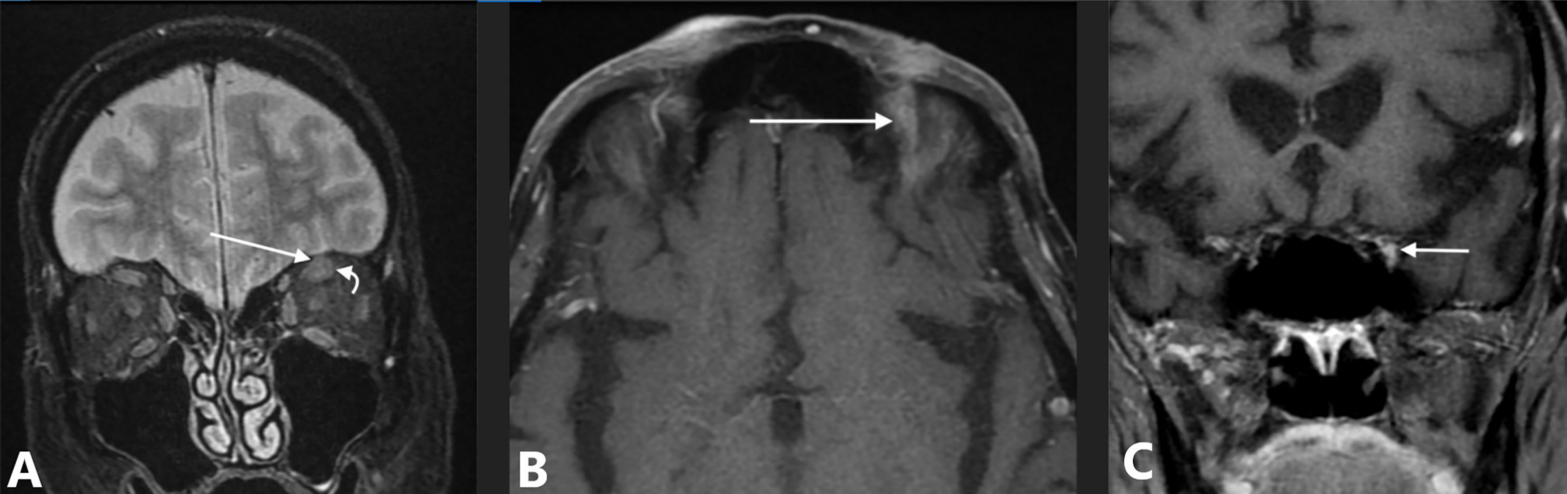
Case 3: an 81-year-old man with squamous cell carcinoma and perineural spread along V1 to the cavernous sinus. **(A)** MRI with coronal fat-suppressed T2-weighted image shows intermediate signal intensity tumor involving V1 (straight arrow) adjacent to normal superior rectus muscle (curved arrow). **(B)** Axial fat-suppressed contrast-enhanced T1-weighted image shows abnormal enhancement in the superior orbit (arrow). **(C)** Coronal contrast-enhanced T1-weighted image, obtained just posterior to the superior orbital fissure, shows asymmetric enhancement and fullness along the lateral margin of the cavernous sinus (arrow).

An 81-year-old man without significant medical problems presented in March 2020 with a cutaneous nodule and severe pain above the left eyebrow at the site of four prior Mohs microsurgery procedures for SCC. Biopsy of the skin lesion revealed moderately to poorly differentiated squamous cell carcinoma with a residual nerve engulfed and surrounded by tumor. MRI of the brain demonstrated a 1.0-cm soft tissue mass at the left superior orbital rim with tumor perineural extension along V1 into the left supraorbital foramen, left orbit, and left cavernous sinus (**Figure 3**). After reviewing treatment options, he decided against surgery due to morbidity and need for enucleation in favor of the ICPi, cemiplimab 350 mg IV every 3 weeks, and by the third infusion, his neurotropic pain had resolved. Soon after the fourth infusion, he developed progressive shortness of breath, and a resting oxygen saturation of 97% decreased to 80% with ambulation. Computerized tomography (CT) scan showed extensive peribronchial parenchyma consolidation in all lung segments, and results of SARS-CoV-2 tests were repeatedly negative. He was initiated on high-dose corticosteroids and hospitalized out of concern for grade 3 immune-mediated pneumonitis. Bronchoalveolar lavage was negative for infection; however, his respiratory status failed to improve with steroids. Empiric antimicrobials and sequential immunosuppressive therapy with anti-tumor necrosis factor-alpha inhibitor and intravenous immune globulin (IVIG) were provided. Upon further clinical decline, the patient required intubation and mechanical ventilation with repeat bronchoalveolar lavage revealing infection with *Pneumocystis jirovecii*. The patient died several days later after his family elected for comfort-directed care.

### Case 4

**Figure 4:**
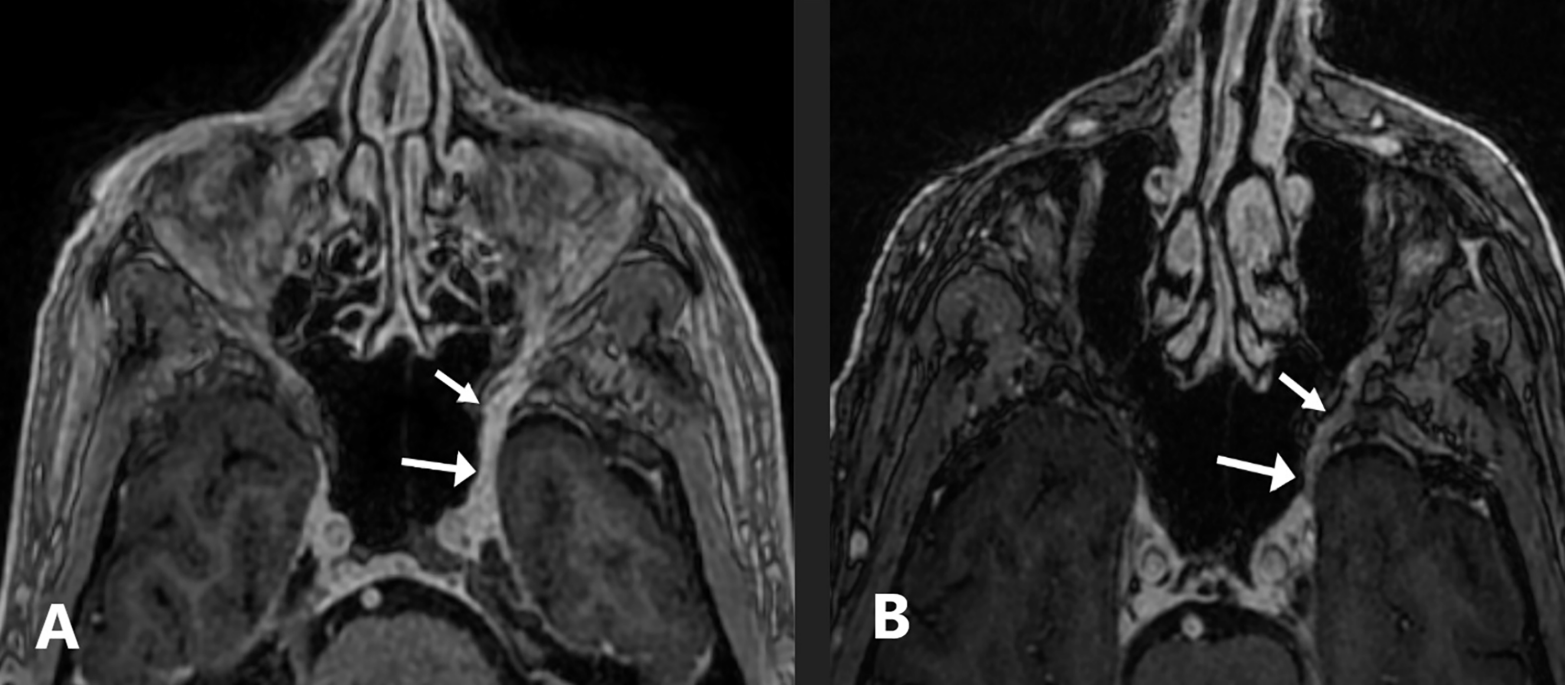
Case 4: a 74-year-old-man with squamous cell carcinoma. **(A)** Axial fat-suppressed contrast-enhanced T1-weighted image shows a thickened, abnormally enhancing left V2 nerve extending from the premaxillary fat to the inferior cavernous sinus and along the left Vidian nerve (arrows). **(B)** Axial fat-suppressed contrast-enhanced T1-weighted image performed 6 months after permanently discontinuing pembrolizumab shows mild asymmetric enhancement of the left V2 nerve that is improved compared to pre-treatment (arrows).

A 74-year-old man with a history of invasive well-differentiated cutaneous SCC of the nose underwent Mohs microsurgery in April 2016 with negative margins after four stages and no mention of PNI. In the fall of 2017, he started to notice some left facial numbness, but this was attributed to recent dental work. The left facial numbness persisted, and in May 2018, the patient presented with left facial droop and noted that the numbness had now spread to the right side of his face. MRI of the brain demonstrated perineural tumor spread along the left V2 segment extending from the premaxillary fat to the inferior cavernous sinus and along the left Vidian nerve (**Figure 4**). A 1.8-cm enhancing subcutaneous mass was also seen along the right infraorbital cheek. Subsequent biopsy of the left infraorbital nerve revealed peripheral nerve tissue with areas of moderately differentiated SCC and associated chronic inflammation. Excision of the right subcutaneous cheek nodule confirmed invasive moderately differentiated SCC with an infiltrative pattern and PNI. The patient declined definitive radiation therapy over concerns for significant toxicity and elected to pursue immunotherapy with pembrolizumab 200 mg IV every 3 weeks citing emphasis on quality of life. His facial numbness did not improve despite radiographic response. After 12 infusions, he developed severe diarrhea and was diagnosed with grade 3 immune-related colitis confirmed on colonoscopy. He was treated with high-dose corticosteroids with rapid resolution of symptoms. Shared decision-making led to immunotherapy rechallenge, but the patient had recurrence of colitis after four infusions, and thus, treatment was permanently discontinued. The patient completed 1 year of pembrolizumab and has been off therapy since September 2019 with clinically stable disease.

## Discussion

Endoneural and perineural metastasis is a common mechanism of aggressive tumor spread in which primary malignancies fan along the course of neural pathways. There is an estimated 1%–5% incidence of PNI in head and neck KCs with cPNI occurring most commonly along the facial and trigeminal nerves (29). Patients presenting with neuropathic symptoms in a region of previously treated KC should prompt clinicians to evaluate for recurrent disease along neural pathways. Patients presenting with facial palsy or trigeminal neuralgia are often misdiagnosed to have benign cranial neuropathies resulting in delayed treatment, increased morbidity, and decreased quality of life. MRI detects perineural spread with the highest specificity, whereas earlier PNI detection within the head and neck has been shown with high-resolution MR neurography protocols (30).

Historically, complete surgical excision of perineural and endoneural metastases was often limited in its success with the extent of surgical resection recommended to include the entire skin regions supplied by the affected nerve (31). Current literature detailing treatment modalities of KCs with PNI stems mostly from treatment of cutaneous squamous cell carcinoma of the head and neck (CSCCHN). Surgical approaches and outcomes have improved since advances in MRI neurography for preoperatively defining the zonal distribution of disease, resulting in 5-year disease-free survival rates ranging between 50% and 75% following excision and post-operative radiation (32). This approach is superior to postoperative concurrent chemoradiotherapy, which has not been shown to provide statistical benefit in overall survival, disease-free survival, or even freedom from locoregional relapse when compared with surgery and postoperative radiotherapy alone (33). There may be a role for adjuvant proton radiotherapy where risk for toxicities following radiation therapy, including retinopathies, optic neuropathies, hearing loss, and brain or brainstem necrosis, may be mitigated (34).

Two ICPis, cemiplimab and pembrolizumab, are Food and Drug Administration (FDA)-approved and now considered the standard of care for locoregionally advanced or metastatic cutaneous SCC in patients who are not eligible for curative surgery or radiation. Data from the Phase II EMPOWER-CSCC-1 study with cemiplimab demonstrated clinically meaningful improvements with an overall response rate (ORR) of 46.1% and complete response (CR) rate of 16.1% at 3-year follow-up (35). The median time to a CR was 11.2 months, and both the duration of response (DOR) and overall survival had not yet been reached. *Post-hoc* exploratory analysis of this cemiplimab study also showed symptomatic benefit with improvements in health-related quality of life and pain control (36). Similarly, the Phase II KEYNOTE-629 study with pembrolizumab reported an ORR of 50% in the locally advanced cohort and 35.2% in the recurrent/metastatic cohort, including a 16.7% and 10.5% CR rate, respectively (37). The median DOR was not reached in either cohort. Both agents induce relatively high response rates with durable disease control. Early phase studies suggest that neoadjuvant immunotherapy may facilitate less extensive upfront treatment (38, 39). Cemiplimab was also recently FDA approved for locally advanced or metastatic BCC previously treated with or inappropriate for a hedgehog inhibitor and is the only ICPi with this indication where responses have shown early evidence of durability in the Phase II Study 1620 (40). The hedgehog inhibitor, vismodegib, has shown promising results as a neoadjuvant strategy in the Phase II VISMONEO study of locally advanced BCC, with ORR of 71% and 25 of 44 patients with pathological assessment of response demonstrating a complete response (41).

As we see through our four cases highlighting variable degrees of clinical PNI, management of patients with symptoms of PNI does not fit a single mold. While ICPi came to be clearly indicated in the first case, the patient’s quality of life worsened during the period of diagnostic evaluation that delayed initiation of therapy. Ultimately, the multi-disciplinary provision of treatment including palliative radiation, ICPi, and chemotherapy provided symptom and disease control; however, he succumbed to infection in the setting of multiple comorbid conditions attributable to his cancer and treatments. The second case demonstrated a delicate balance between the risks and benefits of either intricate surgery or palliative immunotherapy. Due to the availability of extraordinary surgical expertise, the patient was able to undergo curative intent surgery and adjuvant proton radiation with success. The third case illustrates lethal sequelae of immunotherapy-related toxicity in a patient who was eligible for potentially curative resection but declined due to misalignment with his individual goals of care. The last case underscores the durability of response to immunotherapy in the absence of surgery or radiation, although again not without known toxicity risks. All cases bring into question the utility of neo-adjuvant and adjuvant systemic therapies to reduce surgical morbidity and recurrence for high-risk KC, both of which are currently being studied in clinical trials. Whether ICPi could be a practical treatment option for other neurotropic malignancies such as prostate and pancreatic adenocarcinomas remains underexplored. This is primarily due to the immunosuppressive tumor microenvironment that characterizes these “cold tumors” and hence predicts marginal response to ICPi without robust biomarkers. Here, PNI itself may mediate a cold immune microenvironment and signify the need for combinatorial ICPi strategies to overcome inherent treatment resistance (42).

In conclusion, patients presenting with symptoms suspicious for cPNI warrant an expanded diagnostic evaluation to precisely correlate neurological findings with neurotropic spread of disease. While nerve biopsies can be precarious in sensitive areas, a history of skin cancer and clinical presentation suggestive of neurotropism may be enough to pursue timely management in the form of surgery, radiation, and/or systemic therapy given each patient’s individual priorities, comorbidities, and prognosis. It is imperative that the pathology laboratory properly process the nerve specimens and resection specimens to ensure sufficient sectioning that limits sampling error. Among patients considering immunotherapy instead of definitive local therapy, one must consider the potential for immune-related adverse events that may arise from ICPi. A multi-disciplinary review and approach to the management of patients with KC and cPNI is essential given the complexity of therapeutic decision making.

## Ethics Statement

Written informed consent was obtained from the individual(s) for the publication of any potentially identifiable images or data included in this article.

## Author Contributions

Conception and design: GSA and KBB. (II) Administrative support: GSA and KBB. (III) Provision of study materials or patients: AKR, ARS, BH, FK, KBB, SLS, and SJP. (IV) Collection and assembly of data: ARS, BH, GSA, FK, KBB, and SJP. (V) Data analysis and interpretation: AKR, ARS, BH, FK, KBB, SLS, and SJP. (VI) Manuscript writing: AKR, ARS, BH, FK, GSA, KBB, SLS, and SJP. (VII) Final approval of manuscript: AKR, ARS, BH, FK, GSA, KBB, SLS, and SJP. All authors contributed to the article and approved the submitted version.

## Funding

Funding for this manuscript provided by Scripps MD Anderson Cancer Center.

## Conflict of Interest

The authors declare that the research was conducted in the absence of any commercial or financial relationships that could be construed as a potential conflict of interest.

## Publisher’s Note

All claims expressed in this article are solely those of the authors and do not necessarily represent those of their affiliated organizations, or those of the publisher, the editors and the reviewers. Any product that may be evaluated in this article, or claim that may be made by its manufacturer, is not guaranteed or endorsed by the publisher.
